# Sarcopenic Obesity and Risk of Cardiovascular Disease and Mortality: A Population-Based Cohort Study of Older Men

**DOI:** 10.1111/jgs.12652

**Published:** 2014-01-15

**Authors:** Janice L Atkins, Peter H Whincup, Richard W Morris, Lucy T Lennon, Olia Papacosta, S Goya Wannamethee

**Affiliations:** * Department of Primary Care and Population Health, University College LondonLondon, UK; ‡ Population Health Research Centre, Division of Population Health Sciences and Education, St George's, University of LondonLondon, UK

**Keywords:** cardiovascular disease, mortality, muscle mass, obesity, sarcopenia

## Abstract

**Objectives:**

To examine associations between sarcopenia, obesity, and sarcopenic obesity and risk of cardiovascular disease (CVD) and all-cause mortality in older men.

**Design:**

Prospective cohort study.

**Setting:**

British Regional Heart Study.

**Participants:**

Men aged 60–79 years (n = 4,252).

**Measurements:**

Baseline waist circumference (WC) and midarm muscle circumference (MAMC) measurements were used to classify participants into four groups: sarcopenic, obese, sarcopenic obese, or optimal WC and MAMC. The cohort was followed for a mean of 11.3 years for CVD and all-cause mortality. Cox regression analyses assessed associations between sarcopenic obesity groups and all-cause mortality, CVD mortality, CVD events, and coronary heart disease (CHD) events.

**Results:**

There were 1,314 deaths, 518 CVD deaths, 852 CVD events, and 458 CHD events during follow-up. All-cause mortality risk was significantly greater in sarcopenic (HR = 1.41, 95% CI = 1.22–1.63) and obese (HR = 1.21, 95% CI = 1.03–1.42) men than in the optimal reference group, with the highest risk in sarcopenic obese (HR = 1.72, 95% CI = 1.35–2.18), after adjustment for lifestyle characteristics. Risk of CVD mortality was significantly greater in sarcopenic and obese but not sarcopenic obese men. No association was seen between sarcopenic obesity groups and CHD or CVD events.

**Conclusion:**

Sarcopenia and central adiposity were associated with greater cardiovascular mortality and all-cause mortality. Sarcopenic obese men had the highest risk of all-cause mortality but not CVD mortality. Efforts to promote healthy aging should focus on preventing obesity and maintaining muscle mass.

Important changes to body composition occur with age; although body weight and body mass index (BMI) may remain relatively unchanged, typically visceral fat increases and muscle mass decreases.^1^ Sarcopenic obesity refers to the age-associated loss of muscle mass coupled with high levels of adiposity, but no consensus definition of sarcopenic obesity exists.^2^–^4^ Measuring the effect of obesity in elderly adults may be limited when using BMI, because it combines fat and muscle mass,^5^ but abdominal obesity is an established risk factor for cardiovascular disease (CVD) and all-cause mortality. A meta-analysis of 29 elderly cohorts including men and women aged 65–74 years showed a significant positive association between waist circumference (WC) and all-cause and CVD mortality risk that was consistent across BMI categories.^6^

Prospective studies have also shown consistent associations between low muscle mass and mortality risk,^7^–^11^ although the association between sarcopenia and CVD risk is not well established, and the combined effects of sarcopenia and obesity on CVD and all-cause mortality have not been well studied. A recent review found that the majority of studies examining associations between sarcopenic obesity and health outcomes have focused on functional capacity or disability or have been cross-sectional.^12^ Sarcopenic obesity has been linked prospectively to greater risk of all-cause mortality in disease-specific populations,^13^,^14^ but few studies have prospectively examined the effect of sarcopenic obesity on CVD outcomes and mortality. A large longitudinal study, the Cardiovascular Health Study, found that sarcopenic obesity classified using muscle strength was modestly associated with CVD risk but that sarcopenic obesity classified using muscle mass was not associated with CVD risk.^15^

A previous report from this cohort examined anthropometric indexes of body composition and found that high WC and low muscle mass, as measured using midarm muscle circumference (MAMC), were associated with all-cause mortality,^10^ but the concept of sarcopenic obesity was not examined, and the influence of sarcopenia (low muscle mass) on CVD risk was not explored. The objective of this study was to prospectively examine associations between sarcopenia, obesity, and sarcopenic obesity, defined using WC and MAMC measurements, and risk of CVD events and all-cause mortality in a large, nationally representative, population-based study of older British men. A secondary objective was to examine associations between sarcopenia, obesity, and sarcopenic obesity defined using alternative measurements, fat mass (FM) and fat-free mass (FFM), and risk of CVD events and all-cause mortality.

## Methods

### Participants and Study Design

The British Regional Heart Study is a prospective study in a socioeconomically and geographically representative sample of 7,735 British men from 24 towns in Great Britain.^16^ This cohort of predominantly white Europeans (>99%) was initially examined in 1978–1980. Twenty years later, in 1998–2000, 4,252 men (77% of survivors), then aged 60–79, attended a physical examination, provided a fasting blood sample, and completed a questionnaire.^17^ This study used data from the 20-year re-examination and follow-up data on CVD and mortality until 2010. Participants provided written informed consent in accordance with the Declaration of Helsinki. Ethical approval was obtained from all relevant local research ethics committees.

### Anthropometric Measurements and Sarcopenic Obesity Definition

Anthropometric measurements at re-examination included height, weight, WC, midupper arm circumference, triceps skinfold thickness, FM, and FFM as described previously.^10^ Participants were also asked to report whether their weight had changed in the previous 3 years, and a dichotomous weight loss variable was created (yes = weight loss; no = no change, gain, or fluctuation). WC was chosen to indicate abdominal obesity instead of a measure of total obesity. Obesity was defined using an established sex-specific cut-point (WC > 102 cm).^18^ MAMC (cm) was used as a marker of muscle mass and was calculated as midupper arm circumference (cm) –0.3142 ×  triceps skinfold thickness (mm).^19^ MAMC has been shown to correlate strongly with more-accurate measures of lean mass measured using dual energy X-ray absorptiometry.^20^ Because no consensus definition of sarcopenia has yet been adopted,^4^ a standard statistical approach was used to define sarcopenia: participants in the lowest two-fifths of the MAMC distribution. Participants were categorized into four nonoverlapping sarcopenic obesity groups: optimal (WC ≤102 cm, MAMC > 25.9 cm), sarcopenic (WC ≤102 cm, MAMC ≤25.9 cm), obese (WC > 102 cm, MAMC > 25.9 cm), or sarcopenic obese (WC > 102 cm, MAMC ≤25.9 cm).

### Alternative Sarcopenic Obesity Definition

For comparative purposes, an alternative sarcopenic obesity classification was created using FFM and FM measurements, determined using bioelectrical impedance analysis (BIA) on fasting participants (Bodystat 500, Bodystat Ltd, Douglas, UK). FFM was calculated using the Deurenberg equation,^21^ and FM was calculated as body weight (kg) – FFM (kg). FFM and FM measures were normalized for height by dividing by height (m^2^) to give a FFM index (FFMI) and a FM index (FMI) in kg/m^2^.^22^ For comparability with the MAMC and WC categories, participants in the lowest two-fifths of the FFMI were classified as sarcopenic (≤16.7 kg/m^2^), and those above the percentile point of FMI corresponding to the WC obesity cutoff (28.7th percentile) were classified as obese (>11.1 kg/m^2^).

### CVD Risk Factors

Cigarette smoking, physical activity, alcohol intake, and occupational social class were self-reported in a questionnaire, and blood pressure, blood lipids, and lung function (forced expiratory volume in 1 second (FEV_1_)) were measured as described previously.^10^,^16^,^23^,^24^ Men were classified into four cigarette smoking groups (never smoked, long-term ex-smoker, recent ex-smoker, current smoker). A physical activity score was derived on the basis of physical activity frequency and type, and men were grouped into six categories: inactive, occasional (regular walking or recreational activity only), light (more-frequent recreational activities, sporting exercise less than once a week, or regular walking plus some recreational activity), moderate (cycling, very frequent weekend recreational activities plus regular walking, or sporting activity once a week), moderately vigorous (sporting activity at least once a week or frequent cycling, plus frequent recreational activities or walking, or frequent sporting activities only), vigorous (very frequent sporting exercise or frequent sporting exercise plus other recreational activities). Validation of this score has been described previously.^23^ The men were asked about drinking frequency (none, occasional or special occasions, weekend, and daily drinkers) and were asked to provide estimated weekly intake. Based on the combined information of drinking frequency and reported weekly estimate, the men were classified into five groups: none; occasional (<1 U/wk), light (1–15 U/wk), moderate (16–42 U/wk), and heavy (>42 U/wk).^24^ Occupational social class was split into three groups (manual, nonmanual, armed forces) based on the longest-held occupation coded using the Registrar General's classification. At baseline, participants were classified as having prevalent myocardial infarction (MI) or prevalent stroke if they had a previous diagnosis of these events according to medical records or self-report. Plasma concentrations of C-reactive protein (CRP), D-dimer, and von Willebrand factor (vWF) were also measured as detailed elsewhere.^25^

### Follow-Up

Participants were followed prospectively for CVD and all-cause mortality from re-examination (1998–2000) to June 2010. Follow-up has been achieved for 98% of the cohort.^26^ Information on death was collected through National Health Service Central Registers (death certificates coded using the *International Classification of Diseases, Ninth Revision* (ICD-9)). Fatal MI was defined as ICD-9 codes 410–414, fatal stroke as ICD-9 codes 430–438, and fatal CVD as ICD-9 codes 390–459. A nonfatal MI was diagnosed according to World Health Organization criteria.^27^ Nonfatal stroke events were those that produced a neurological deficit that was present for more than 24 hours. Evidence regarding nonfatal MI and nonfatal stroke was obtained according to ongoing general practitioner reports and biennial medical record reviews.^17^ The four outcome measures assessed in this study were coronary heart disease (CHD) events (fatal or nonfatal MI), CVD events (nonfatal MI, nonfatal stroke, or fatal CVD), CVD mortality, and all-cause mortality.

### Statistical Analysis

Of the 4,252 men attending re-examination, 69 with prevalent heart failure were excluded because of exceptionally high mortality rates and the strong association between heart failure and weight loss. In addition, 71 men with missing MAMC or WC data and one who died on the examination day were excluded, leaving 4,111 for analyses. Comparative analysis was performed in 4,045 individuals with BIA measurements, classifying sarcopenic obesity using FMI and FFMI. CRP and D-dimer were log-transformed because distributions were highly skewed. Associations between sarcopenic obesity groups and outcome measures were examined using Cox proportional hazards regression, comparing the sarcopenic, obese, and sarcopenic obese groups with the optimal reference group. Models were adjusted for potential confounders and mediators in a sequential manner, including age (Model 1); lifestyle variables (smoking status, alcohol intake, physical activity, and occupational social class; Model 2); prevalent MI or stroke and cardiovascular risk factors (Model 3); markers of inflammation, coagulation, and endothelial dysfunction (Model 4); and weight loss (Model 5). Smoking status, alcohol intake, occupational social class, physical activity, prevalent MI, prevalent stroke, and weight loss were fitted as categorical variables. Age, high-density lipoprotein cholesterol (HDL-C), systolic blood pressure (SBP), FEV_1_, CRP, D-dimer, and vWF were fitted as continuous variables. An interaction between sarcopenia and obesity was tested for using a Cox proportional hazards model with an interaction term between obesity and sarcopenia (fitted as binary variables using the cut-points described earlier).

Sensitivity analysis excluding men with prevalent MI or stroke (n = 640) was also conducted, but this made no real difference to the direction or magnitude of observed associations (results not shown). Prevalent cases of MI and stroke were therefore included in the analysis, and prevalent MI and prevalent stroke were added as covariates to the models. All statistical analyses were performed using Stata 12.0 (Stata Corp, College Station, TX).

## Results

There were 1,314 deaths during the mean follow-up of 11.3 years, 518 of which were attributed to cardiovascular causes; 458 men experienced a CHD event, and 852 experienced a CVD event. One thousand four hundred ninety (36.2%) men were classified as having optimal WC and MAMC, 1,443 (35.1%) were sarcopenic only, 983 (23.9%) were obese only, and 195 (4.7%) were sarcopenic obese. Participants had a mean age of 68.7 ± 5.5 years, WC of 97.1 ± 10.4 cm, BMI of 26.9 ± 3.6 kg/m^2^, and MAMC of 26.5 ± 2.3 cm. Table1 shows the baseline characteristics of participants according to sarcopenic obesity groups. The optimal reference group was generally at the lowest risk of CVD, and the sarcopenic obese group had the highest proportion of inactive individuals; the highest mean CRP, D-dimer, and vWF levels; and the lowest mean FEV_1_. The sarcopenic group had the greatest proportion of current smokers and self-reported weight loss and the highest mean HDL-C. The obese group had the highest proportion of heavy drinkers, the highest mean SBP, and the lowest mean HDL-C.

**Table 1:** Baseline Characteristics of British Regional Heart Study Participants According to Sarcopenic Obesity Groups (Defined According to Waist Circumference (WC) and Midarm Muscle Circumference (MAMC))

Characteristic	Sarcopenic Obesity Groups^a^				*P*-Value^b^
	Optimal, n = 1,490 (36.2%)	Sarcopenic, n = 1,443 (35.1%)	Obese, n = 983 (23.9%)	Sarcopenic Obese, n = 195 (4.7%)	
Sociodemographic and lifestyle variables					
Age, mean±SD	67.6 ± 5.3	70.0 ± 5.6	68.2 ± 5.3	70.3 ± 5.5	.08
Current smokers, n (%)	157 (10.5)	240 (16.7)	101 (10.3)	26 (13.5)	<.001
Heavy drinkers, n (%)	28 (1.9)	44 (3.1)	37 (3.9)	6 (3.2)	.03
Physically inactive, n (%)	86 (6.0)	154 (11.0)	151 (15.9)	37 (20.2)	<.001
Manual workers, n (%)	691 (46.5)	725 (50.4)	547 (55.7)	113 (58.0)	<.001
Anthropometrics					
WC, cm, mean ± SD	94.2 ± 5.5	90.0 ± 7.6	109.4 ± 6.7	108.6 ± 6.9	<.001
Body mass index, kg/m^2^, mean ± SD	26.4 ± 2.0	24.2 ± 2.5	31.0 ± 3.1	29.2 ± 2.9	<.001
MAMC, cm, mean ± SD	27.6 ± 1.3	24.2 ± 1.3	28.6 ± 1.8	24.7 ± 1.0	<.001
Weight loss in past 3 years, n (%)	194 (13.7)	234 (17.6)	137 (14.9)	27 (15.3)	.04
Biological measures					
High-density lipoprotein, mm, mean ± SD	1.3 ± 0.3	1.4 ± 0.4	1.2 ± 0.3	1.3 ± 0.3	<.001
Systolic blood pressure, mmHg, mean ± SD	148.3 ± 23.4	148.0 ± 25.6	152.2 ± 23.3	149.5 ± 23.2	.001
Forced expiratory volume in 1 second, L, mean ± SD	2.7 ± 0.6	2.5 ± 0.7	2.5 ± 0.6	2.3 ± 0.7	.01
C-reactive protein, mg/L, geometric mean (IQR)^c^	1.4 (0.7–2.7)	1.6 (0.7–3.4)	2.3 (1.2–4.0)	2.8 (1.4–5.6)	<.001
D-dimer, ng/mL, geometric mean (IQR)^c^	76.0 (45.0–114.0)	90.6 (51.0–141.0)	83.2 (50.0–121.0)	106.1 (59.5–155.0)	.003
von Willebrand factor, IU/dL, mean ± SD	132.0 ± 43.2	143.0 ± 46.6	141.9 ± 45.9	156.9 ± 53.0	<.001

SD = Standard Deviation; IQR = Interquartile Range.

a Optimal (WC ≤ 102 cm, MAMC > 25.9 cm); sarcopenic (WC ≤ 102 cm, MAMC ≤ 25.9 cm); obese (WC > 102 cm, MAMC > 25.9 cm); sarcopenic obese (WC > 102 cm, MAMC ≤ 25.9 cm).

b *P*-Value for difference between groups (x^2^ for percentages; analysis of variance for means).

c Log-transformed values.

**Figure 1:**
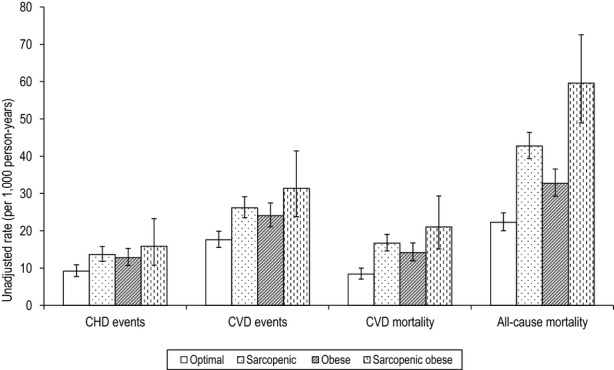
Unadjusted rates of coronary heart disease (CHD) events, cardiovascular disease (CVD) events, CVD mortality, and all-cause mortality (per 1,000 person-years; 95% confidence interval) according to sarcopenic obesity groups (defined according to waist circumference (WC) and midarm muscle circumference (MAMC)). Optimal (WC ≤ 102 cm, MAMC > 25.9 cm), sarcopenic (WC ≤ 102 cm, MAMC ≤ 25.9 cm), obese (WC > 102 cm, MAMC > 25.9 cm), sarcopenic obese (WC > 102 cm, MAMC ≤ 25.9 cm).

Unadjusted rates of all outcomes (CHD events, CVD events, CVD mortality, all-cause mortality) were lowest in the optimal reference group and highest in the sarcopenic obese group, but the difference between these two groups was nonsignificant for CHD events (Figure1). Table2 shows adjusted hazard ratios (HRs) for CHD events, CVD events, CVD mortality, and all-cause mortality by sarcopenic obesity groups. In the age-adjusted model, only the obese group had a significantly greater risk of CHD events than the optimal reference group, but this became nonsignificant after adjusting for lifestyle variables. Sarcopenia, obesity, and sarcopenic obesity were associated with a significantly higher risk of CVD events than in the optimal reference group, but these associations became nonsignificant after adjustment for lifestyle variables.

**Table 2:** Coronary Heart Disease (CHD) Events, Cardiovascular Disease (CVD) Events, CVD Mortality, and All-Cause Mortality According to Sarcopenic Obesity Groups (Defined According to Waist Circumference (WC) and Midarm Muscle Circumference (MAMC))

		Sarcopenic Obesity Groups^a^				*P-*Value (sarcopenia x Obesity Interaction)
Optimal	Sarcopenic	Obese	Sarcopenic obese			
HR	HR (95% CI)	HR (95% CI)	HR (95% CI)			
CHD events (n = 458)	Model 1	1.00	1.22 (0.97–1.53)	1.33 (1.04–1.70)^b^	1.37 (0.90–2.08)	.50
		Model 2	1.00	1.09 (0.86–1.38)	1.19 (0.92–1.55)	1.11 (0.71–1.75)	.55
CVD events (n = 852)	Model 1	1.00	1.19 (1.01–1.41)^b^	1.30 (1.08–1.56)^b^	1.39 (1.02–1.89)^b^	.55
		Model 2	1.00	1.11 (0.93–1.32)	1.18 (0.98–1.43)	1.08 (0.77–1.52)	.31
CVD mortality (n = 518)	Model 1	1.00	1.48 (1.19–1.85)^b^	1.60 (1.25–2.03)^b^	1.79 (1.19–1.85)^b^	.21
		Model 2	1.00	1.35 (1.07–1.70)^b^	1.39 (1.07–1.80)^b^	1.38 (0.91–2.08)	.20
		Model 3	1.00	1.33 (1.04–1.70)^b^	1.18 (0.89–1.55)	1.29 (0.83–2.00)	.44
		Model 4	1.00	1.26 (0.98–1.61)	1.12 (0.84–1.48)	1.14 (0.73–1.79)	.42
All-cause mortality (n = 1314)	Model 1	1.00	1.54 (1.34–1.76)^b^	1.41 (1.21–1.64)^b^	2.09 (1.67–2.62)^b^	.79
		Model 2	1.00	1.41 (1.22–1.63)^b^	1.21 (1.03–1.42)^b^	1.72 (1.35–2.18)^b^	.95
		Model 3	1.00	1.37 (1.18–1.59)^b^	1.11 (0.93–1.32)	1.61 (1.25–2.08)^b^	.70
		Model 4	1.00	1.34 (1.15–1.56)^b^	1.07 (0.90–1.28)	1.49 (1.15–1.93)^b^	.81
		Model 5	1.00	1.34 (1.15–1.57)^b^	1.07 (0.89–1.28)	1.44 (1.10–1.90)^b^	.96

CHD = Coronary Heart Disease; CVD = Cardiovascular Disease; HR = Hazard Ratio; MAMC = Midarm Muscle Circumference; WC = Waist Circumference. Model 1: adjusted for age. Model 2: adjusted for model 1 + smoking, alcohol, occupational social class, physical activity. Model 3: adjusted for model 2 + prevalent MI, prevalent stroke, HDL, SBP, FEV_1._ Model 4: adjusted for model 3 + CRP, D-dimer, vWF. Model 5: adjusted for model 4 + weight loss.

a Optimal (WC ≤ 102 cm, MAMC > 25.9 cm); sarcopenic (WC ≤ 102 cm, MAMC ≤ 25.9 cm); obese (WC > 102 cm, MAMC > 25.9 cm); sarcopenic obese (WC > 102 cm, MAMC ≤ 25.9 cm).

b *P* < .05.

Sarcopenic men and obese men had a higher CVD mortality risk than men with optimal WC and MAMC, and the risk was greatest in the sarcopenic obese men, adjusting for age. After adjustment for lifestyle variables, the greater risks in sarcopenic and obese men remained significant, but the greater risk in sarcopenic obese men was no longer significant. Additional adjustment for prevalent MI, prevalent stroke, HDL-C, SBP, and FEV_1_ resulted in a nonsignificant association in the obese group. Further adjustment for CRP, D-dimer, and vWF in the sarcopenic group attenuated the associations, which became nonsignificant.

Sarcopenic and obese men had significantly greater risk of all-cause mortality after adjustment for lifestyle variables, with the highest risk seen in sarcopenic obese men (sarcopenic, HR = 1.41, 95% CI = 1.22–1.63; obese, HR = 1.21, 95% CI = 1.03–1.42; sarcopenic obese, HR = 1.72, 95% CI = 1.35–2.18). The greater mortality associated with sarcopenia and sarcopenic obesity (but not obesity) remained after adjustment for prevalent MI, prevalent stroke, HDL-C, SBP, FEV_1_, CRP, D-dimer, vWF, and weight loss (sarcopenic, HR = 1.34, 95% CI = 1.15–1.57; sarcopenic obese, HR = 1.44, 95% CI = 1.10–1.90). There was no evidence of interaction between sarcopenia and obesity for any of the outcomes.

Risk of non-CVD mortality was significantly greater in sarcopenic men (HR = 1.39, 95% CI = 1.14–1.70) and sarcopenic obese men (HR = 1.73, 95% CI = 1.23–2.42) than in the optimal group, even after adjustment, but a significantly greater risk in non-CVD mortality was not seen in the obese group.

Table3 shows adjusted HRs for outcomes according to sarcopenic obesity groups using the alternative classification of FFMI and FMI measurements. After adjustment for lifestyle variables (Model 2) there was no significantly greater risk of any outcomes in the sarcopenic, obese, or sarcopenic obese group than in the optimal reference group.

**Table 3:** Coronary Heart Disease (CHD) Events, Cardiovascular Disease (CVD) Events, CVD Mortality, and All-Cause Mortality According to Sarcopenic Obesity Groups (Defined According to Fat Mass Index (FMI) and Fat-Free Mass Index (FFMI))

		Sarcopenic Obesity Groups^a^				*P-*Value (sarcopenia x Obesity Interaction)
Optimal	Sarcopenic	Obese	Sarcopenic Obese			
HR	HR (95% CI)	HR (95% CI)	HR (95% CI)			
Total, n (%)		1670 (41.3)	1190 (29.4)	756 (18.7)	429 (10.6)	
CHD events (n = 450)	Model 1	1.00	1.17 (0.94–1.46)	1.13 (0.87–1.48)	1.35 (1.00–1.83)^b^	.92
	Model 2		1.00	1.15 (0.91–1.45)	1.04 (0.79–1.38)	1.13 (0.82–1.56)	.79
CVD events (n = 827)	Model 1	1.00	1.05 (0.89–1.24)	1.10 (0.90–1.33)	1.21 (0.97–1.51)	.77
		Model 2	1.00	1.03 (0.87–1.22)	1.01 (0.82–1.24)	1.01 (0.79–1.29)	.85
CVD mortality (n = 502)	Model 1	1.00	1.18 (0.95–1.46)	1.43 (1.12–1.82)^b^	1.37 (1.02–1.82)^b^	.29
		Model 2	1.00	1.11 (0.89–1.39)	1.25 (0.96–1.62)	1.11 (0.81–1.53)	.30
All-cause mortality (n = 1288)	Model 1	1.00	1.19 (1.05–1.36)^b^	1.26 (1.08–1.47)^b^	1.18 (0.98–1.42)	.05
		Model 2	1.00	1.08 (0.94–1.24)	1.09 (0.92–1.27)	0.98 (0.80–1.20)	.19

CHD = Coronary Heart Disease; CVD = Cardiovascular Disease; FFMI = Fat-Free Mass Index; FMI = Fat Mass Index; HR = Hazard Ratio. Model 1: adjusted for age. Model 2: adjusted for model 1 + smoking, alcohol, occupational social class, physical activity.

a Cutoff for obesity (FMI > 28.7th percentile) corresponds to the WC > 102cm cutoff. Optimal (FMI ≤ 11.1 kg/m^2^, FFMI > 16.7 kg/m^2^); sarcopenic (FMI ≤ 11.1 kg/m^2^, FFMI ≤ 16.7 kg/m^2^); obese (FMI > 11.1 kg/m^2^, FFMI > 16.7 kg/m^2^); sarcopenic obese (FMI > 11.1 kg/m^2^, FFMI ≤ 16.7 kg/m^2^)

b *P* < .05.

## Discussion

The current study investigated the effects of sarcopenia, obesity, and sarcopenic obesity on CVD and mortality risk in a prospective cohort of older men, adding to the limited literature in this area. Sarcopenic obesity was classified using two methods, and the role of a wide range of potential risk factors, including inflammatory and hemostatic markers, that had not previously been explored was examined. Sarcopenia (MAMC ≤ 25.9 cm) and obesity (WC > 102 cm) were associated with CVD mortality and all-cause mortality risk. Sarcopenic obese men had the highest risk of all-cause mortality but did not have an excess risk of CVD mortality beyond that associated with sarcopenia or obesity alone. Comparative analysis, using FFMI and FMI measurements to classify sarcopenic obesity, found no significant associations between sarcopenic obese groups and any of the outcomes.

### WC and MAMC Measurements

Sarcopenia, obesity, and sarcopenic obesity were not associated with significantly greater risk of CHD events or CVD events after adjustment for lifestyle variables, although sarcopenia was associated with CVD mortality even after adjustment for blood pressure and blood lipids. This association was no longer significant after adjustment for CRP, D-dimer, and vWF, suggesting that inflammation explained this greater CVD mortality, which is consistent with the finding that CRP is more strongly associated with the risk of fatal vascular events than nonfatal vascular events.^28^ Obesity was associated with greater risk of CVD mortality, which was attenuated after adjustment for established cardiovascular risk factors. The observed association between abdominal obesity and greater risk of CVD mortality is consistent with a previous meta-analysis in elderly adults.^6^ Cross-sectionally, sarcopenic obese men had the least favorable cardiovascular risk profile, which is consistent with other cross-sectional studies,^29^,^30^ but sarcopenic obesity was not associated with greater CVD mortality risk after adjustment for lifestyle variables, perhaps because of the small numbers in this group. The findings of the current study are broadly consistent with those of a prospective study of community-dwelling older men and women (aged ≥65) that found that the risk of CHD and CVD events was not significantly greater in the sarcopenic, obese, or sarcopenic obese groups (as determined according to WC and BIA-measured muscle mass) than in the optimal group after adjustment for behavioral variables,^15^ although the prior study did not specifically examine CVD mortality, which was associated with sarcopenia and obesity in the current study. The authors of this aforementioned study implied that muscle strength rather than muscle mass may be more important, because sarcopenic obesity (defined using grip strength) was predictive of greater risk of CVD events.

Sarcopenia was associated with greater all-cause mortality, which was independent of lifestyle and cardiovascular risk markers. This is consistent with previous prospective studies in older adults that have found associations between various measures of low muscle mass and greater mortality risk.^7^–^9^,^11^ Although inflammation is strongly related to sarcopenia and all-cause mortality,^31^,^32^ these previous studies did not assess the contributing role of CRP; the current study showed that inflammation did not explain the association between sarcopenia and mortality. Obesity was associated with greater all-cause mortality, independent of lifestyle variables, but the association disappeared after adjustment for established cardiovascular risk factors. Despite obesity being a strong risk factor for mortality, some previous studies have shown that overweight and obesity are not as adverse in elderly populations.^5^,^33^,^35^ The results of the current study are also consistent with a prospective study suggesting that muscle mass (measured using midupper arm circumference) may have a stronger association with mortality than obesity (measured using BMI).^11^ Sarcopenic obese older men had a higher risk of all-cause mortality than the optimal reference group after adjustment for lifestyle variables. The observed association between sarcopenic obesity and mortality diminished slightly after adjustment for potential mediators (blood pressure, blood lipids, and inflammation), but significantly greater risk remained, suggesting that cardiovascular and inflammatory risk markers only partially explain the relationship between sarcopenic obesity and mortality. Moreover, sarcopenic obesity was more strongly related to non-CVD mortality, independent of inflammation, than CVD mortality. Despite the sarcopenic obese group having the highest risk of mortality, there was no evidence of interaction between sarcopenia and obesity, suggesting that the presence of obesity does not modify the effect of sarcopenia (or equivalently, that the presence of sarcopenia not modify the effect of obesity).

This study confirms initial work performed in this cohort suggesting that the combined use of WC and MAMC provides simple anthropometric body composition measures to assess the risk of mortality in older men.^10^ The current study has almost double the follow-up (period extended from 6 to 11 years) and includes additional outcomes (CHD events, CVD events, and CVD mortality). The results are consistent with the limited evidence from prospective studies on the association between sarcopenic obesity and mortality in disease-specific states^13^,^14^ and extend findings to a large sample drawn from an older general population. The current study found a direction of association between sarcopenic obesity and all-cause mortality similar to that of a previous study with longer follow-up in which overweight men below the first tertile of grip strength had 1.39 times the mortality risk as normal-weight men above the third tertile.^36^

### Fat Mass and Fat-Free Mass Measurements

Using FMI and FFMI measurements to classify sarcopenic obesity, there was no significant difference in risk of outcomes between the sarcopenic, obese, or sarcopenic obese groups and the optimal reference group after adjustment for behavioral variables. This supports previous research in this cohort suggesting that a composite anthropometric measure of MAMC and WC is more effective in predicting all-cause mortality than measures of FFMI and FMI.^10^ These null results are also consistent with a previous study showing that the risk of CHD and CVD events was not higher in sarcopenic or sarcopenic obese individuals using BIA-measured muscle mass.^15^ The use of BIA to assess FFM in elderly adults can be inaccurate principally because of the variability that exists in FFM hydration.^37^,^38^ This may explain the observed lack of association between BIA-defined sarcopenic obesity and outcomes seen here.

### Strengths and Limitations

The strengths of this study were that it was a large population-based cohort with high follow-up levels and that two different muscle mass measures were compared, although older men, predominantly of white European ethnic origin, were investigated, so findings may not be generalizable to women and nonwhite groups. Although all study outcomes were based on objective measurements, self-reported variables may have been subject to misclassification. Residual confounding may have existed, for example in the case of physical activity, which was measured subjectively. Observed associations between sarcopenic obesity groups and outcomes may have been further attenuated if objective measures of physical activity had been available. A direct measure of adiposity or muscle mass such as computed tomographic scanning or magnetic resonance imaging was not available, but such expensive, time-consuming measures are rarely available in primary care settings, and MAMC and WC represent a practical alternative. The cutoff used for the lowest two-fifths of MAMC (≤25.9 cm) was comparable with that used in another population-based study of men (aged ≥80) that used a cutoff below the first tertile of 21.1 cm.^9^ WC has also been shown to be the anthropometric variable that best correlates with adiposity stores as measured by magnetic resonance imaging, in men.^39^ Using imprecise measurements of adiposity and muscle mass may have attenuated the strength of associations observed between sarcopenic obesity groups and outcomes. The European Working Group on Sarcopenia in Older People has suggested defining sarcopenia using muscle mass and function (strength and performance).^4^ Measures of muscle function were not available here, so findings are applicable to sarcopenic obesity as defined by muscle mass but not function.

In conclusion, sarcopenia and abdominal obesity are associated with all-cause mortality, with the highest risk in sarcopenic obese men. Sarcopenia and sarcopenic obesity, but not obesity on its own, were associated with greater all-cause mortality independent of CVD risk factors, inflammation, and weight loss. No association was found between sarcopenia and obesity and CHD and CVD events, but sarcopenia and obesity were associated with greater CVD mortality, largely because of their associations with blood pressure, blood lipids and inflammation. Efforts to promote healthy aging should focus on preventing obesity and maintaining muscle mass.
